# Ventricular Tachycardia Ablation in Patients with Left Ventricular Assist Devices

**DOI:** 10.19102/icrm.2019.101101

**Published:** 2019-11-15

**Authors:** Kaustubha D. Patil, Jonathan Chrispin

**Affiliations:** ^1^Division of Cardiology, Department of Medicine, Johns Hopkins University School of Medicine, Baltimore, MD, USA

**Keywords:** Catheter ablation, electroanatomical mapping, left ventricular assist device, ventricular tachycardia

## Abstract

In this complex case study, we discuss a patient who underwent successful catheter ablation for ventricular tachycardia following left ventricular assist device placement. We discuss the technique and review existing literature in an effort to explore the feasibility and safety of this procedure in this clinical setting.

## Introduction

Because of limited organ availability, the number of heart transplantations performed per year is plateauing.^1^ Thus, the use of mechanical support devices—in particular, left ventricular assist devices (LVADs)—for the treatment of end-stage heart failure has increased. However, it is well-recognized that ventricular arrhythmia (VA) burden may actually rise following the implantation of an LVAD,^2^ which is associated with a decrease in patient survival.^3^ Catheter ablation for recurrent, drug-refractory ventricular tachycardia (VT) in patients with structural heart disease is well-established,^4–6^ resulting in fewer appropriate implantable cardioverter-defibrillator (ICD) shocks and repeat hospitalizations. In this report, we discuss the case of a patient with drug-refractory VA following LVAD placement that was successfully mapped and terminated with catheter ablation.

## Case presentation

A 68-year-old male with a finding of ischemic cardiomyopathy after undergoing HeartMate™ II LVAD (Abbott Laboratories, Chicago, IL, USA) implantation in October 2013 presented to the Johns Hopkins Hospital with one week of worsening fatigue and palpitations as well as multiple ICD shocks on the morning of admission. His previous history included implantation of a dual-chamber ICD, prior ablation of scar-mediated VT, persistent atrial fibrillation, and amiodarone-induced thyrotoxicosis.

**Figure 1:**
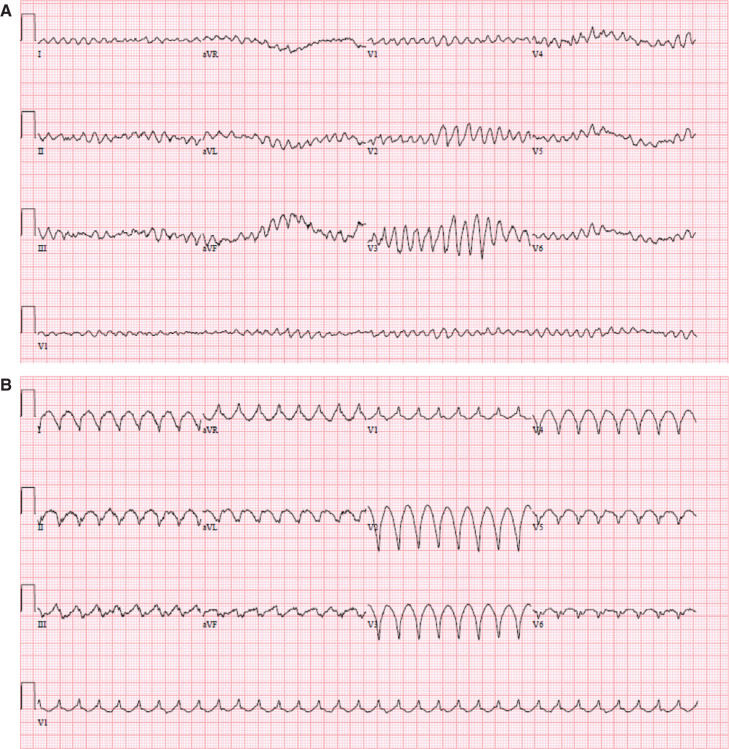
**A:** An electrocardiogram taken upon initial presentation shows VF, which was hemodynamically tolerated by the patient given his LVAD and which required external defibrillation. **B:** Later during his hospital stay, the patient exhibited recurrent monomorphic VT.

The patient’s rhythm upon presentation to the emergency department was ventricular fibrillation (VF), which was hemodynamically tolerated, given his LVAD **(Figure 1A)**. He underwent successful external defibrillation with one shock of 200 J and a restoration of sinus rhythm.

ICD interrogation showed a prolonged episode (more than five hours) of tachycardia. Atrial lead electrograms were consistent with atrial fibrillation (atrial cycle length: 180–210 ms). Ventricular lead electrograms were consistent with a simultaneous, regular VT with a ventricular cycle length of approximately 280 ms to 290 ms. His device appropriately detected VT, and an ICD shock converted his rhythm to polymorphic VT/VF. There were then five subsequent additional shocks that failed to terminate the polymorphic VT/VF.

He was admitted to the cardiac intensive care unit, where he experienced recurrent sustained monomorphic VT despite intravenous lidocaine, propanolol, and sotalol **(Figure 1B)**. The electrocardiogram morphology of his VT showed right bundle branch block, a rightward axis, and an indeterminate vertical axis (negative in II, isoelectric in III, and negative in aVF) suggestive of an apical lateral left ventricular origin. The VT was subsequently successfully terminated with antitachycardia pacing via his ICD. With the LVAD introduced, all VT episodes were hemodynamically stable, but the patient complained of palpitations.

**Figure 2:**
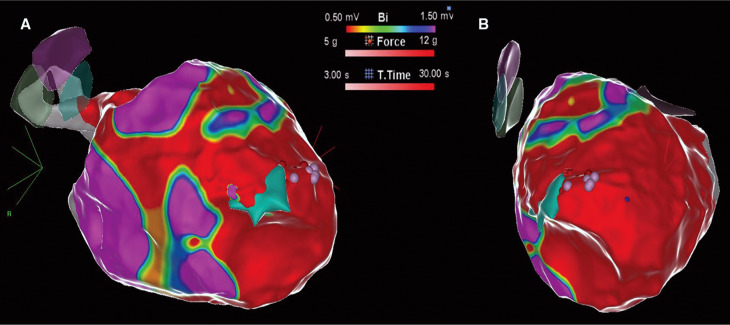
Electroanatomical map in the **(A)** right anterior oblique and **(B)** left anterior oblique orientations of the left ventricle with extensive dense scar (in red).

Given the patient’s recurrent VT requiring ICD therapy despite antiarrhythmic drug therapy, he was referred for an electrophysiology study and catheter ablation. An endocardial shell of the left ventricle, left ventricular outflow tract, and LVAD inflow cannula insertion site was created using a CARTO-Sound^®^ (Biosense Webster, Diamond Bar, CA, USA) intracardiac echocardiogram catheter. Endocardial left ventricular mapping was performed using CARTO^®^ (Biosense Webster, Diamond Bar, CA, USA) by way of a single transseptal approach and using a DECA-NAV^®^ (Biosense Webster, Diamond Bar, CA, USA) mapping catheter. Endocardial left ventricular mapping was notable for a large anterior wall scar from the base to the apex, spanning from the septum to the lateral wall **(Figures 2A and 2B)**. The apical segments of the scar just lateral to the LVAD inflow cannula insertion site had multiple sites of slow conduction with isolated late potentials and low-amplitude, long-fractionated signals.

**Figure 3:**
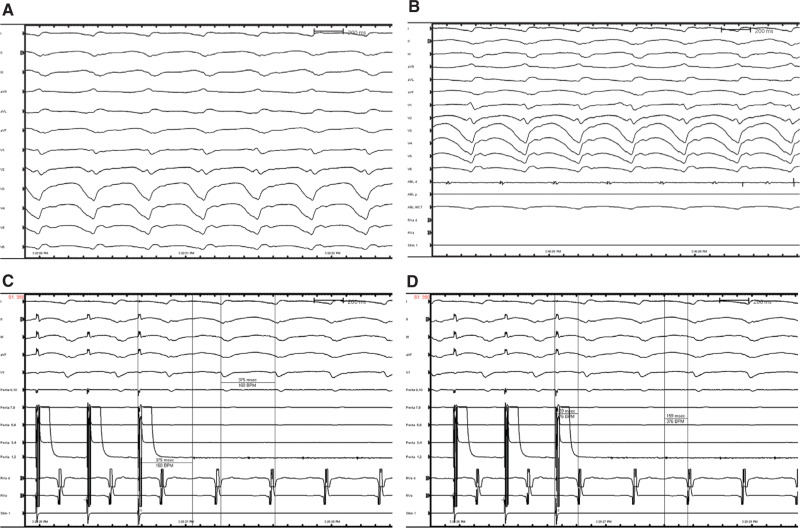
**A:** A 12-lead electrocardiogram during the electrophysiology study shows easily inducible monomorphic VT. **B:** Intracardiac electrograms on the ablation distal (ABL d) catheter shows fractionated middiastolic potentials. **C:** Entrainment from this location shows concealed entrainment with a short postpacing interval consistent with the site of pacing being in the critical isthmus. **D:** The stimulus-to-QRS interval length equals the electrogram-to-QRS interval length with a timing that is 42% of the TCL, consistent with the central isthmus.

**Figure 4:**
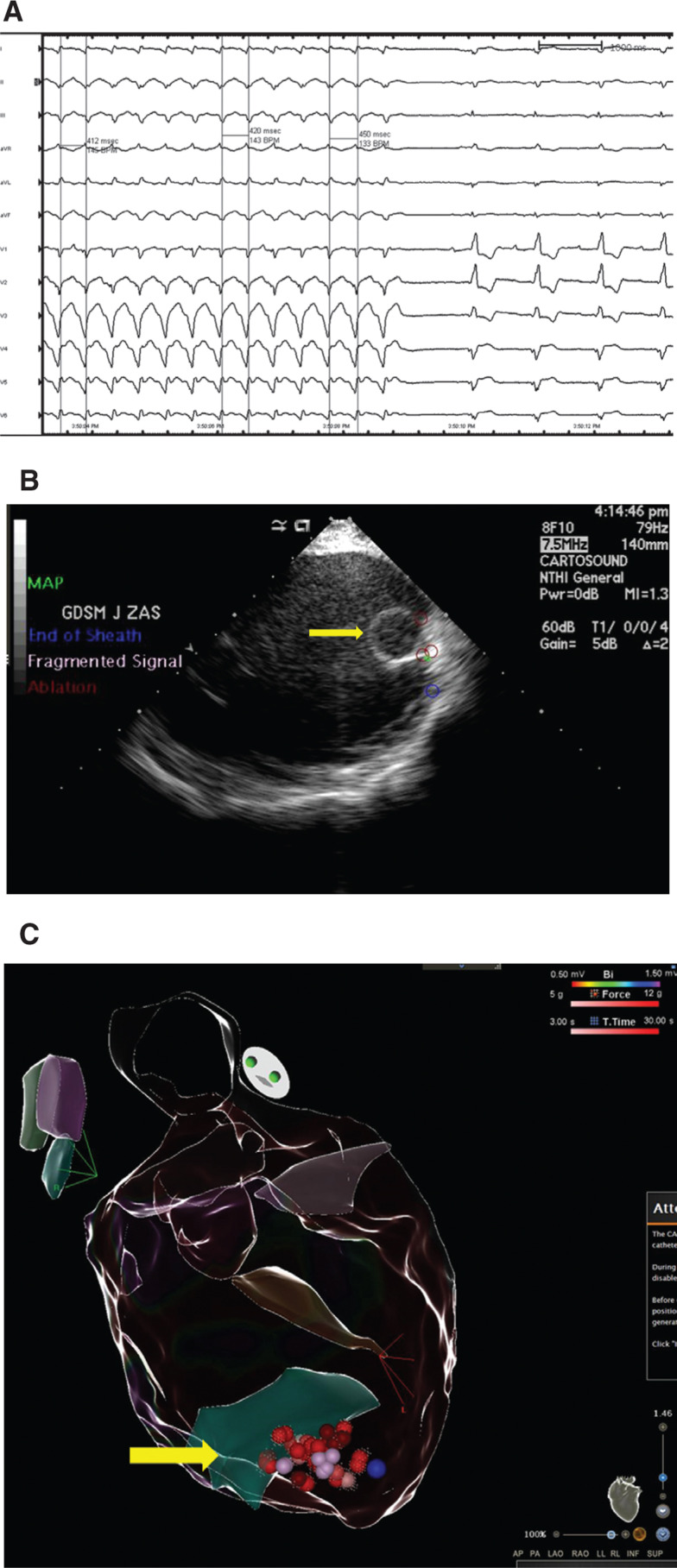
**A:** During ablation in the critical isthmus, the TCL was prolonged before termination. **B:** Intracardiac echocardiogram imaging shows the ablation lesions (red circles) near the LVAD cannula (yellow arrow). **C:** Electroanatomical map depicting the ablation lesions (red balls) near the LVAD inflow cannula (yellow arrow).

On isoproterenol, the patient’s clinical VT was induced **(Figure 3A)**. During VT, the mentioned sites of slow conduction showed middiastolic potentials **(Figure 3B)**. Entrainment mapping from these sites was concealed with a postpacing interval that closely approximated the tachycardia cycle length (TCL) **(Figure 3C)**. The stimulus-to-QRS interval length was equal to the electrogram-to-QRS interval length **(Figure 3D)**. The stimulus-to-QRS interval length (159 ms) as compared with the TCL (375 ms) was consistent with a critical isthmus site being at this location (ie, stimulus to-QRS length/TCL ratio of 42%). Using an irrigated, force-sensing ablation catheter, radiofrequency ablation lesions were delivered to the sites consistent with a critical isthmus, showing slowed ventricular conduction and subsequent termination of VT during ablation **(Figure 4A)**. The successful ablation site was visualized with intracardiac echocardiography **(Figure 4B)**. Additional substrate-based ablation was performed to connect the critical isthmus sites to the apical scar **(Figure 4C)**. Postablation, there was no inducible VT with ventricular programmed stimulation, either on or off isoproterenol. Given the documented rapid atrial rates on initial device interrogation, we proceeded with atrioventricular node ablation for the definitive rate control of rapid atrial arrhythmias. The atrioventricular node was successfully ablated, with resultant complete heart block and pacing dispensed via the patient’s previously implanted ICD.

Given the multiple failed ICD shocks administered for polymorphic VT/VF after the prolonged VT prior to presentation, defibrillation threshold testing was performed. A 50-Hz pulse was delivered by the patient’s internal defibrillator, resulting in polymorphic VT/VF. The patient’s dual-chamber ICD appropriately detected VF and delivered an appropriate shock of one shock of 35 J, which successfully restored normal sinus rhythm. There were no intraoperative complications, and the patient did well postprocedure without the recurrence of the presenting clinical VT. He was discharged on the fifth day after ablation on a regimen of propranolol, sotalol, and mexiletine.

## Discussion

VAs are common among patients with structural heart disease, and ICDs have been shown to improve survival in both primary and secondary prevention populations.^7,8^ However, despite various advances that have been made in ICD technology, ICD shocks are associated with a decrease in long-term survival and with a decline in quality of life.^9^ Antiarrhythmic therapy is effective in decreasing VAs, but these medications can be associated with significant side effects.

Elsewhere, while LVAD therapy has emerged as a promising option to improve survival in patients with end-stage heart failure, it has been repeatedly reported that LVADs may actually increase the VA burden.^4^ In a single-center retrospective analysis of 43 patients who underwent implantation of a HeartMate™ II LVAD (Abbott Laboratories, Chicago, IL, USA), 27.9% experienced early electrical storm, defined as three or more ICD therapy applications in 24 hours, with a median time from LVAD implant of 9.1 days ± 7.8 days. Those who had early VT also had a significantly higher all-cause mortality rate at 30 days (33.3% versus 6.5%).^2^ These findings were replicated in a larger study of 98 patients with ICDs who underwent placement of an LVAD. In total, 48 (49%) of the 98 patients experienced VA and experienced an average of 30 ± 98 appropriate ICD therapy applications.^10^ One of the main predictors of VA post-LVAD implantation is a history of pre-LVAD VA.^2,10,11^ Our patient had a history of VA prior to his LVAD placement.

Unfortunately, successful elimination of VA post-LVAD placement remains difficult despite antiarrhythmic medications and, thus, catheter ablation has emerged as an adjunct to decrease the VA burden. Ideally, invasive activation and entrainment mapping during VT is the most specific way to identify the critical isthmus that contributes to the maintenance of monomorphic VT. However, in most circumstances, mapping in VT is not feasible, given that VT is not hemodynamically tolerated, particularly in a substrate with poor systolic function and advanced heart failure. Decreased end-organ perfusion results in significant metabolic acidosis, leading to an increase in periprocedural complications and mortality. A substrate-based approach of identifying areas of slow conduction in sinus rhythm has emerged as an alternative strategy.

One of the benefits of mechanical hemodynamic support during catheter ablation of VA is that one can maintain organ perfusion during VA, allowing for more detailed and precise electroanatomical mapping of the VA circuits. Patients with LVADs often have hemodynamically tolerable VAs, as seen in our patient who presented with VF. Baratto et al. reported on 64 patients who underwent catheter ablation of unstable VA with extracorporeal membrane oxygenation (ECMO) for hemodynamic support. At least one VT was terminated in 81% of the procedures performed and, more importantly, the study demonstrated the safety of ECMO-supported VA catheter ablation.^12^ Herweg et al. presented their center’s experience of six patients regarding the feasibility of catheter ablation of VA in patients with LVAD. In two of the six patients, the patients’ inflow cannula served as the substrate for the VA.^5^

Sacher et al. reported a multicenter experience of patients who underwent catheter ablation of VA in the presence of a HeartMate™ II LVAD (Abbott Laboratories, Chicago, IL, USA). In total, 34 patients with a mix of nonischemic and ischemic cardiomyopathies, respectively, underwent 39 ablation procedures.^13^ In this series, nine patients required VA ablation at less than 30 days after their LVAD placement secondary to refractory VT. Ultimately, only 9% (10/110) of the targeted VT episodes within the study population were related to the LVAD cannula. In a meta-analysis of 18 studies on the catheter ablation of VA post-LVAD implantation, 90.3% of the VAs were found to be of scar-related reentry origin, while 19.3% were related to the LVAD cannula.^6^ Following successful catheter ablation, the VT storm terminated in 90% of patients and there was a significant reduction in ICD therapy applications as compared with prior to surgery (23.8% versus 57.1%). Activation/entrainment mapping was performed in 61.2% of the patients. Further, despite the presence of an aortic outflow tract cannula, which limits access into the ventricule, 36.4% of the procedures were performed using a retrograde aortic approach.

In conclusion, our case demonstrated the feasibility and safety of activation/entrainment mapping in a patient with scar-related VT. In the setting of a large area of anterior scar, ablation was limited to a small area, allowing for a precision-oriented ablation strategy, further decreasing the potential for complications associated with prolonged procedures and ablation times. Of note, our patient had a VT circuit involving the LVAD inflow cannula, which is seen in only a minority of patients with VT post-LVAD. The use of CARTO-Sound^®^ (Biosense Webster, Diamond Bar, CA, USA) allowed for real-time geometry creation and visualization of the inflow cannula throughout the procedure. The present case supports that catheter ablation for drug-refractory VT in patients with LVADs is feasible and should be included in the armamentarium of treatment strategies in specialized cardiac centers.
